# Quantitative proteomic analysis of super soft kernel texture in soft white spring wheat

**DOI:** 10.1371/journal.pone.0289784

**Published:** 2023-08-31

**Authors:** Meriem Aoun, Jose M. Orenday-Ortiz, Kitty Brown, Corey Broeckling, Craig F. Morris, Alecia M. Kiszonas

**Affiliations:** 1 Department of Crop and Soil Sciences, Washington State University, Pullman, Washington, United States of America; 2 Department of Entomology and Plant Pathology, Oklahoma State University, Stillwater, Oklahoma, United States of America; 3 Firestone Pacific Foods, Vancouver, Washington, United States of America; 4 Formerly School of Food Science, Washington State University, Pullman, Washington, United States of America; 5 Analytical Resources Core, Colorado State University, Fort Collins, Colorado, United States of America; 6 USDA-ARS Western Wheat & Pulse Quality Laboratory, Washington State University, Pullman, Washington, United States of America; University of Delhi, INDIA

## Abstract

Super soft kernel texture is associated with superior milling and baking performance in soft wheat. To understand the mechanism underlying super soft kernel texture, we studied proteomic changes between a normal soft and a super soft during kernel development. The cultivar ‘Alpowa’, a soft white spring wheat, was crossed to a closely related super soft spring wheat line ‘BC2SS163’ to produce F_6_ recombinant inbred lines (RILs). Four normal soft RILs and four super soft RILs along with the parents were selected for proteomic analysis. Alpowa and the normal soft RILs showed hardness indices of 20 to 30, whereas BC2SS163 and the super soft RILs showed hardness indices of -2 to -6. Kernels were collected from normal soft and super soft genotypes at 7 days post anthesis (dpa), 14 dpa, 28 dpa, and maturity and were subject to quantitative proteomic analysis. Throughout kernel development, 175 differentially abundant proteins (DAPs) were identified. Most DAPs were observed at 7 dpa, 14 dpa, and 28 dpa. Of the 175 DAPs, 32 had higher abundance in normal soft wheat, whereas 143 DAPs had higher abundance in super soft wheat. A total of 18 DAPs were associated with carbohydrate metabolism and five DAPs were associated with lipids. The gene *TraesCS4B02G091100*.*1* on chromosome arm 4BS, which encodes for sucrose-phosphate synthase, was identified as a candidate gene for super soft kernel texture in BC2SS163. This study enhanced our understanding of the mechanism underlying super soft kernel texture in soft white spring wheat.

## Introduction

Kernel texture or grain hardness is the primary determinant of wheat (*Triticum aestivum L*.) milling and baking quality [1, 2]. Grain hardness determines wheat end-use as hard wheat is used for making pan breads, whereas soft wheat is used for making crackers, cookies, cakes, steam breads, and some Asian-style noodles [3, 4]. The puroindoline genes (*Pina-D1* and *Pinb-D1*) at the *Hardness* (*Ha*) locus on chromosome arm 5DS [5–7] are the primary factors controlling grain hardness and distinguishing soft wheat from hard wheat. The presence of puroindoline wild-type genes results in soft texture, whereas the absence or mutation in the puroindoline genes results in harder kernels [8]. Grain hardness is also influenced by various physical and chemical factors including kernel size, vitreousness, protein content, water-soluble pentosans (non-starch polysaccharides), and lipid content [2, 9, 10].

A unique kernel texture described as ‘super soft’ was characterized by lower grain hardness indices compared to ‘normal soft’ wheat [11–15]. Using the Single Kernel Characterization System (SKCS), super soft wheat lines show grain hardness indices as low as negative values compared to normal soft lines that typically have grain hardness indices of ∼20-35. The SKCS crushes individual kernels and uses an algorithm that accounts for weight and moisture to produce a unitless hardness index. The algorithm was built for U.S. soft common wheat in which hardness indices were centered on a value of 25 and for hard common wheat in which hardness indices were centered on a value of 75. However, there are no specific limits to the SKCS scale, so negative values can be achieved [11]. Super soft kernel texture was reported to improve end-use quality of soft white wheat food products as it is associated with higher break flour yield, lower water absorption, and larger cookie diameters [11].

Super soft kernel phenotype is independent of the puroindolines as both super soft wheat and normal soft wheat carry the wild-type genes. Previous genetic studies identified large effect genomic loci associated with super soft kernel texture [12, 13, 15, 16]. In soft white spring wheat, Kumar et al. [12, 13] performed a quantitative trait loci (QTL) analysis on a recombinant inbred line (RIL) population developed from a cross between a super soft line ‘BC2SS163′ and the normal soft wheat cultivar ‘Alpowa’ (PI 566596) and identified one major locus on chromosome arm 4BS. Using genome wide association studies, Aoun et al. [15] identified two major loci on chromosomes 5A and 3A associated with super soft kernel texture in soft white winter wheat. Ibba et al. [16] identified two major loci on chromosomes 1B and 4B associated with super soft texture in a soft durum wheat RIL population derived from a cross between the super soft durum wheat (*T*. *turgidum* ssp. *durum*) variety ‘Soft Strongfield’ and the Khorasan durum wheat variety (*T*. *turgidum* ssp. *turanicum*) ‘KAMUT®’.

Advances in mass spectrometry have allowed proteomic analysis studies in diverse organisms [17–21]. A fully annotated wheat genome sequence [22] has enabled a thorough characterization of wheat at the proteome level using modern mass spectrometry technologies [23]. These breakthroughs facilitated our study to characterize proteins associated with the super soft kernel texture in wheat. The objective of this study was to investigate differentially abundant proteins (DAPs) between normal soft and super soft kernel texture during kernel development. This investigation will enhance our understanding of the mechanism underlying the super soft phenotype.

## Materials and methods

### Plant material

Eight soft white spring wheat F_6_ RILs (9–02, 5–12, 9–14, 4–05, 8-6-1, 5-4-1, 1-10-5, and 5-4-3) were developed through single seed descent from a cross between the soft white spring wheat parent Alpowa and a closely related super soft spring wheat line BC2SS163. Alpowa is derived from the cross Fielder/Potam-70//Wal-laday/3/Walladay/Potam-70 [24] and BC2SS163 was derived from the cross Alpowa/SS163/2* Alpowa [12, 13]. Therefore, the parental lines of this cross were theoretically 87.5% identical. The eight RILs were selected based on their grain hardness indices, where four lines showed SKCS hardness similar to Alpowa, whereas the remaining four lines showed SKCS hardness similar to BC2SS163 (Table 1). The parents and RILs were grown at the Plant Growth Facility, Washington State University, Pullman, WA. A total of 14 plants (replicates) per line were grown and anthesis dates were recorded for each plant.

**Table 1:** Grain hardness of super soft and normal soft wheat lines.

Group	Line	Generation ^a^	Grain hardness	LSD test ^b^
**Normal Soft**	Alpowa	Parental line	20.3	b
	9–02	F6 RIL	30.2	b
	5–12	F6 RIL	25.1	b
	9–14	F6 RIL	23.4	b
	4–05	F6 RIL	22.4	b
**Super soft**	BC2SS163	Parental line	-2.1	a
	8-6-1	F6 RIL	-2.9	a
	5-4-1	F6 RIL	-4.8	a
	1-10-5	F6 RIL	-2.0	a
	5-4-3	F6 RIL	-5.7	a

^a^ RIL: recombinant inbred line

^b^ Fisher’s Least Significant Difference (LSD) test. Lines with different letters are significantly different at 95% level of confidence.

Kernels were collected from each RIL plant at 7-days post anthesis (dpa), 14 dpa, 28 dpa, and maturity. For the parental lines, only mature kernels were acquired. Kernel samples collected from the RILs at different developmental stages, except maturity, were lyophilized and kept at -80°C until processed. Mature kernels of the parents and the RILs were dried, and grain hardness indices (unitless) were measured based on 200 kernels per sample using SKCS 4100 (Perten Instruments, North America, Inc., Springfield, IL, USA) according to the AACCI Approved Method 55–31 [25]. For each kernel development stage, kernels from three replicates (plants) per line were pooled and a sample was acquired for proteomic analysis. Therefore, we had a total of 34 samples for proteomics, (i.e., eight RILs × four development stages + 2 samples for the parents at maturity). For analysis, the four RILs from the super soft group and the four RILs from the normal soft group were considered as replicates at each harvest date. The parents were included in the analysis only at maturity.

### Protein extraction and quantification

Samples were weighed in 5mL bead beating tubes (Axygen) with a target weight of 130 mg/sample. Except for 7 dpa, kernels were initially crushed using the stomper (NextAdvance; 20 strokes). Six or seven 3.5 mm stainless steel UFO beads (NextAdvance) were then added to each sample followed by bead beating in a Bullet Blender 5 Storm (NextAdvance) at speed 10 for 5 min. Lysis buffer containing 1% Universal Nuclease (Easy Pep 96 MS Sample prep Kit, ThermoFisher Scientific) was added at a 3.5:1 buffer : sample ratio followed by bead beating at speed 12 for 5 min. Homogenate was then transferred to 1.5 mL microcentifuge tube and cup horn sonicated on ice (amplitude 70, 10s pulse followed by 20s rest; 9 min total sonication). Debris was pelleted via centrifugation at 16,000×g at 4°C for 10 minutes. Small supernatant aliquots were diluted 1:10 in 2M urea, 2% SDS and measured for total protein content using the Pierce BCA Protein Assay Kit (ThermoFisher Scientific) following manufacturer instructions. Absorbance at 550 nm was measured on a BioRad 680 microplate reader and total protein concentrations were calculated based on a bovine serum albumin standard curve fit to a quadratic (Microplate Manager 5 software, BioRad).

### Trypsin digestion

Extracted proteins were processed using the EasyPep 96 MS Sample Prep Kit (ThermoFisher Scientific) following the manufacturer’s instructions. Briefly, 110 μg total protein was aliquoted from each sample and raised to 100 μl with lysis buffer. Reduction and alkylation solutions were sequentially added with gentle mixing followed by incubation at 95˚C for 10 min. After cooling to room temperature, 5 μg of a Trypsin/LysC mixture was added and samples were digested with shaking at 37°C for 3 hours. The enzymes were then deactivated with the digestion stop solution and contaminants removed using mixed mode peptide clean up resin in 96 well plate format. Peptide eluates were transferred to microcentrifuge tubes, dried in a vacuum evaporator and then resuspended in 5% acetonitrile (ACN), 0.1% formic acid (FA). Once resolubilized, absorbance at 205 nm was measured using a NanoDrop (ThermoScientific) and total peptide concentration was then calculated using an extinction coefficient of 31 [26].

### Tandem mass tag labeling

A total of 25 μg peptide from each sample (or MixQC) was aliquoted and raised to 75 μl using triethylammonium bicarbonate. All Tandem Mass Tag (TMT) label reagents were allowed to equilibrate to room temperature followed by addition of 41 μl LC-MS acetonitrile and occasional vortexing over 5 min. All vials of like labels were then pooled together and mixed via vortex. A volume of 31 μl of TMT label was added to each sample and incubated at room temperature for 1 hour. Quenching was achieved by addition of hydroxylamine (0.37% final) and 15 min additional incubation. Following quenching, samples were pooled into 9 TMT sets. Pooled peptides were then acidified to pH <3 using 80 μl 10% trifluoroacetic acid prior to using high pH reverse phase fractionation spin columns for desalting, removal of excess TMT label, and fractionation of peptides.

### Peptide fractionation

A total of 100 μg total peptide was subjected to fractionation using spin columns from the Pierce High pH Reversed-Phase Peptide Fractionation Kit following manufacturer’s instructions for TMT labeled peptides. The wash solution consisted of 0.1% Triethylamine (TEA) as diluent and ACN added to 5% final concentration. Two washes were performed. Elution solutions contained 0.1% TEA as diluent with ACN added to 10–50% final concentration. Stepwise fractionation took place using 300 μl elution solution and spinning at 3000×g for 2 min at room temperature. A total of eight fractions were collected and subsequently dried in a Savant speedvac, reconstituted in 15 μl of 5% ACN, 0.1% FA, and quantified using the NanoDrop as described above.

### Mass spectrometry (MS) analysis

The eight fractions from each TMT set were block randomized and injected in a randomized set order. A total of 0.75 μg of peptides were purified and concentrated using an on-line enrichment column (Waters Symmetry Trap C18 100Å, 5μm, 180 μm ID x 20 mm column). Chromatographic separation was performed at a flow rate of 350 nanoliters/min on a reverse phase nanospray column (Waters, Peptide BEH C18; 1.7 μm, 75 μm ID x 150 mm column, 45°C) using a 85 min linear gradient from 5%-40% buffer B (99.9% ACN, 0.1% FA) followed by 40–85% buffer B over 7 min. Peptides were eluted directly into the mass spectrometer (Orbitrap Velos Pro, Thermo Scientific) equipped with a Nanospray Flex ion source (Thermo Scientific) and spectra were collected over a m/z range of 400–1500, positive mode ionization. The top 10 ions with charge state +2 or higher were accepted for MS/MS using a dynamic exclusion limit of 1 MS/MS spectra of a given m/z value for 30 s (exclusion duration of 120s). The instrument was operated in FT profile mode detection for both MS and MS/MS detection (resolution of 30,000 and 7,500 respectively). Fragmentation was via higher energy collision dissociation (HCD) with a normalized collision energy set to 35%. Xcalibur 3.0 software (Thermo Scientific) was used to create compound lists of the spectra with a S/N threshold of 1.5 and 1 scan/group.

### Protein identification and quantitation

Tandem mass spectra were acquired, charge state deconvoluted, and deisotoped using ProteoWizard MsConvert version 3.0. Spectra from all samples were searched using Mascot (Matrix Science, London, UK; version 2.6.0) against reverse concatenated Uniprot*Triticum aestivum* reference proteome (UP000019116 downloaded 11 Nov 2019), GluPro version 1.0 downloaded 12 Nov 2019, and cRAP downloaded 5 October 2018 assuming the digestion enzyme trypsin. Mascot was searched using a parent ion tolerance of 20 ppm and a fragment ion mass tolerance of 0.020 Da. Carbamidomethylation of cysteine and TMT6plex of lysine and the n-terminus were specified in Mascot as fixed modifications. Asparagine and glutamine deamidation and methionine oxidation were specified in Mascot as variable modifications.

Search results from each TMT set were subjected to Multidimensional Protein Identification Technology (MuDPIT), imported, and combined using the probabilistic protein identification algorithms [27] implemented in the Scaffold Q+S software (version Scaffold_5.0.0, Proteome Software Inc., Portland, OR) [28]. Peptide thresholds were set (80%) such that a peptide false discovery rate (FDR) of 0.11% was achieved based on hits to the reverse database [29]. Protein identification was accepted if it could be established at greater than 98.0% probability (1.0% FDR) and contained at least two identified peptides. Protein probabilities were computed using the Protein Prophet algorithm [30]. Proteins with similar peptides and that could not be distinguished using MS/MS analysis were grouped to satisfy the principles of parsimony.

Channels were corrected using values supplied by ThermoScientific (Lots VK313393 and VL313586) according to the algorithm described in Shadforth et al. [31]. Normalization was performed iteratively on intensities across samples and spectra [32]. Means were used for averaging. Using an adaptive intensity weighting algorithm, the spectra were log-transformed, pruned of those corresponding to multiple proteins and those missing a reference value, and weighted. Of 326,453 spectra in the experiment at the given thresholds, 210,732 (65%) were included in quantitation.

### Statistical and bioinformatic analysis

Analysis of variance (ANOVA) was performed for each protein using mixed linear model in lme4 R package [33, 34]. The model was written as

$$Y_{ijk}\;=\;\mu \;+\;{\text{Class}}_{i}\;+\;{\text{Stage}}_{j}\;+\;\text{Class}\;\times \;{\text{Stage}}_{ij}\;+\;{\text{Line}}_{k}\;+\;e_{ijk}$$

where Y = protein log_2_ intensity values, μ = the overall mean (intercept), Class*_i_* = super soft and normal soft wheat class, Stage*_j_* = kernel development stages which are 7 dpa, 14 dpa, 28 dpa and maturity, Class × Stage*_ij_* = wheat class and kernel development stage interaction, Line_k_ = the wheat line effect, and e*_ijk_* = the residual variance. All effects were considered fixed except line which was considered random. Then for each protein, the R function ‘emmeans’ was used for pairwise comparisons to compare protein abundance between normal soft and super soft wheat at different kernel development stages. The default tukey’s option in emmeans function was used to control false discovery for all pairwise tests within a given ANOVA.

All identified proteins in this study (n = 4,324 proteins, S1 Table in S1 File) were used in the identification of differentially abundant proteins (DAPs). Proteins with pairwise comparison *P* value ≤ 0.05 and |log_2_ fold change of protein intensity| ≥ 2 were considered DAPs. Subsequently, Gene Ontology (GO) analysis for DAPs was performed. GO terms and their description were extracted from the iwgsc_refseqv1.0 FunctionalAnnotation_v1__HCgenes_v1.0 gff3 file. GO enrichment analysis for the DAPs at each development stage was performed using the website-based tool AgriGO v2.0 [35] at http://systemsbiology.cau.edu.cn/agriGOv2/specises_analysis.php?SpeciseID=22&latin=Triticum_aestivum/. Resulting GO terms with FDR ≤ 0.05 were considered significantly enriched.

## Results

### Grain hardness

The parental lines had contrasting hardness indices. Alpowa had a hardness index of 20.3 (normal soft wheat) and BC2SS163 had hardness index of -2.1 (super soft wheat) (Table 1).

Four of the RILs had low hardness indices (-5.7 to -2.0) like that observed in BC2SS163 and were classified as super soft wheat lines. The remaining four RILs had hardness indices of 22.4- 30.2 like that observed in Alpowa and were classified as normal soft wheat lines (Table 1).

### Differentially abundant proteins between normal soft and super soft wheat

**Fig 1:**
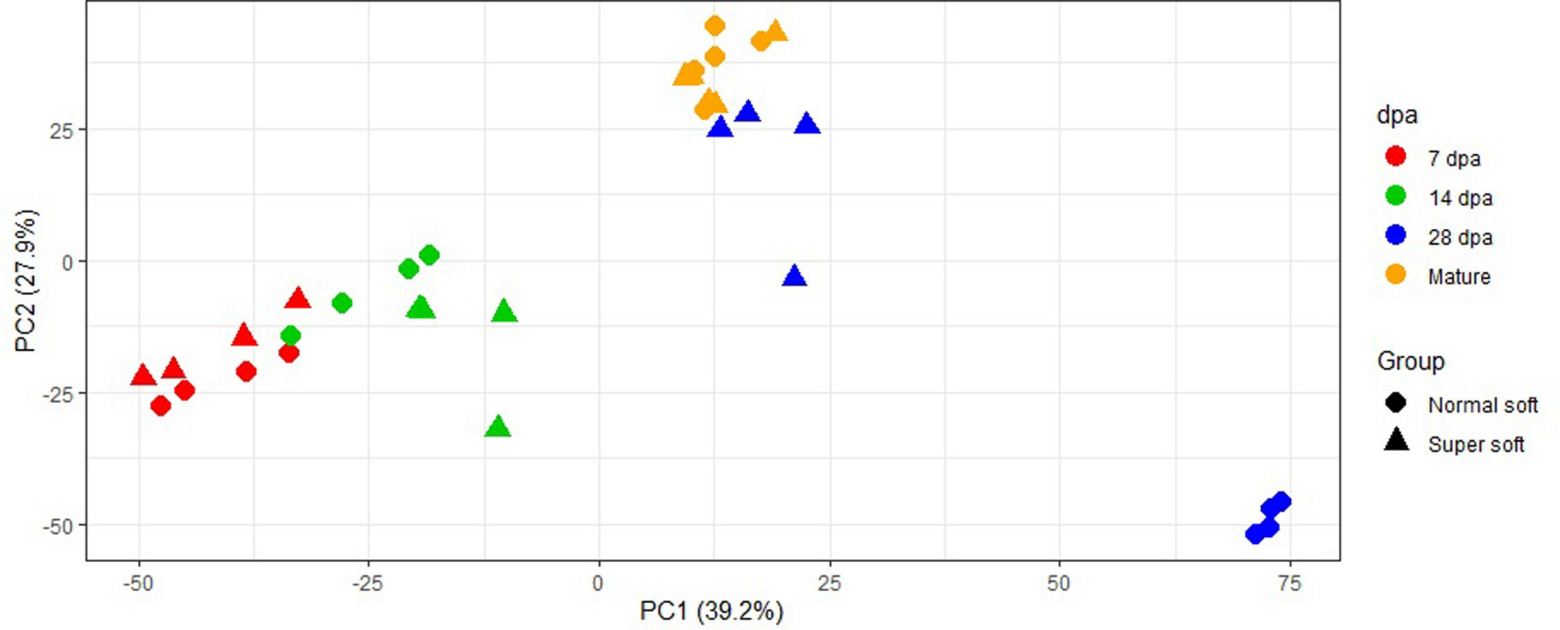
Principal component analysis obtained using 3,242 proteins with missing data < 20% in super soft and normal soft white wheat lines at different kernel development stages. Principal component (PC) analysis based on the first two PCs, PC1 and PC2, which explained 39.2% and 27.9% of the variation, respectively. The different colors correspond to the different kernel developmental stages which are 7 days post anthesis (dpa), 14 dpa, 28 dpa and maturity.

A total of 4,324 proteins were identified in the samples. Log_2_ (protein intensity) in each line throughout kernel development are presented in S1 Table in S1 File. Principal component analysis (PCA) based on the proteome (3,242 proteins with < 20% missing data) showed clustering based on kernel development stages. Clustering was also observed based on wheat line class (normal soft vs. super soft) at most stages except at maturity. The clearest separation between normal soft and super soft was found at 28 dpa (Fig 1). The first two PCs explained cumulatively 67.1% of variation in the proteome.

**Fig 2:**
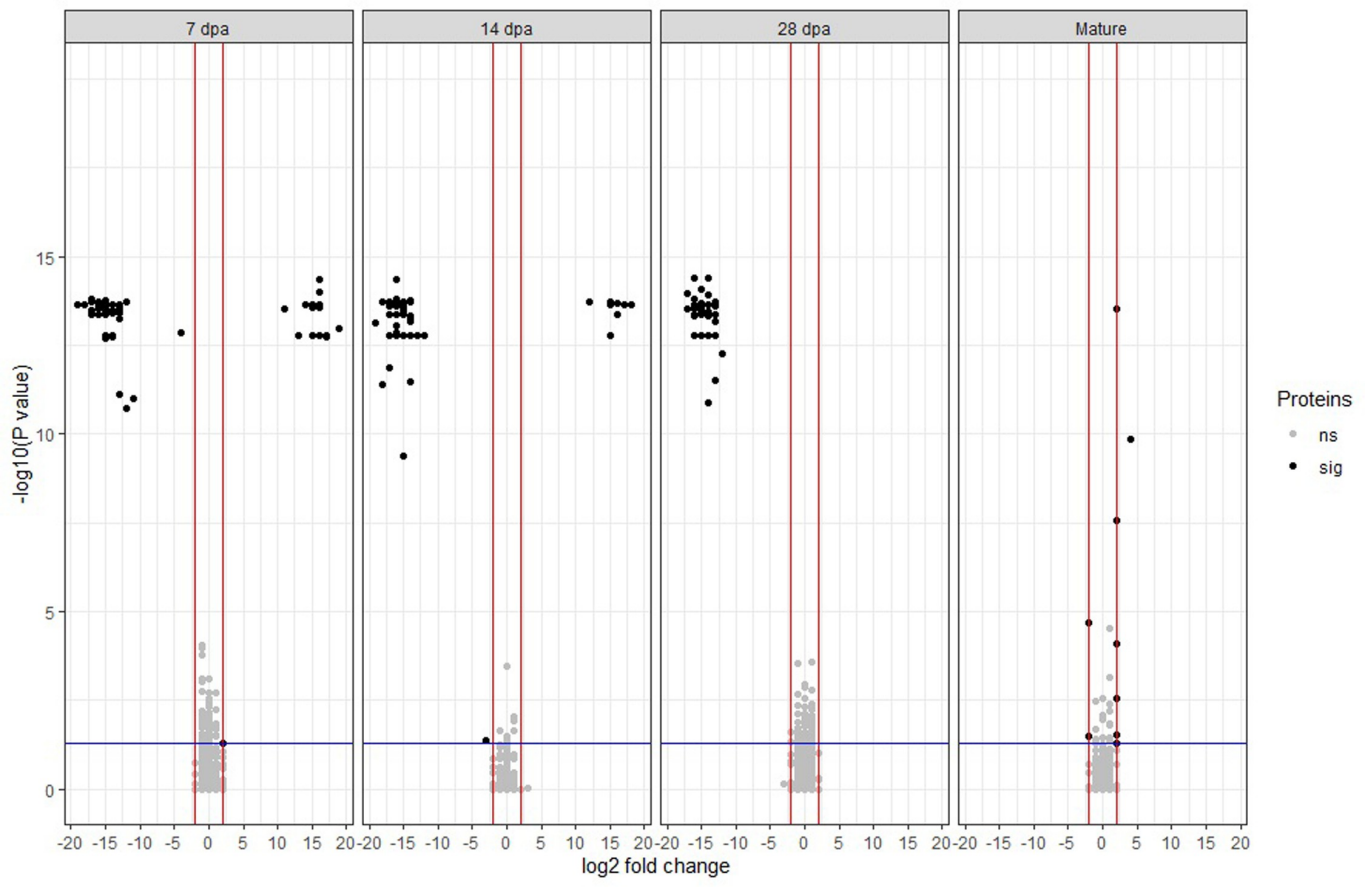
Volcano plots of log_2_ fold change in protein intensity between normal soft and super soft wheat versus -log10 (*P* value) of Tukey’s test for different kernel development stages. Black points correspond to 175 differentially abundant proteins (sig) with *P* value ≤ 0.05 represented with the blue horizontal line and |log2 fold change| ≥ 2 represented with the red vertical lines. Grey points correspond to proteins that are not significant (ns). The kernel development stages are 7 days post anthesis (dpa), 14 dpa, 28 dpa, and maturity.

Using a pairwise comparison of *P* value ≤ 0.05 and |log_2_ fold change of protein intensity| ≥ 2, a total of 175 proteins were differentially abundant (Fig 2 and S2 and S3 Tables in S1 File). Most of these DAPs were observed at 7 dpa, 14 dpa, and 28 dpa.

Based on International Wheat Genome Sequencing Consortium (IWGSC) gene annotation, among these 175 DAPs, there were 18 proteins annotated with functions related to carbohydrate metabolism and five proteins with functions related to lipids. Lipid-associated DAPs include lipid transfer protein, lecithin-cholesterol acyltransferase-like 1, GDSL esterase/lipase, Sn1-specific diacylglycerol lipase alpha, and GDSL esterase/lipase. All these lipid-associated DAPs had higher abundance in super soft wheat with four of them differentially abundant at 28 dpa (S4 Table in S1 File).

**Fig 3:**
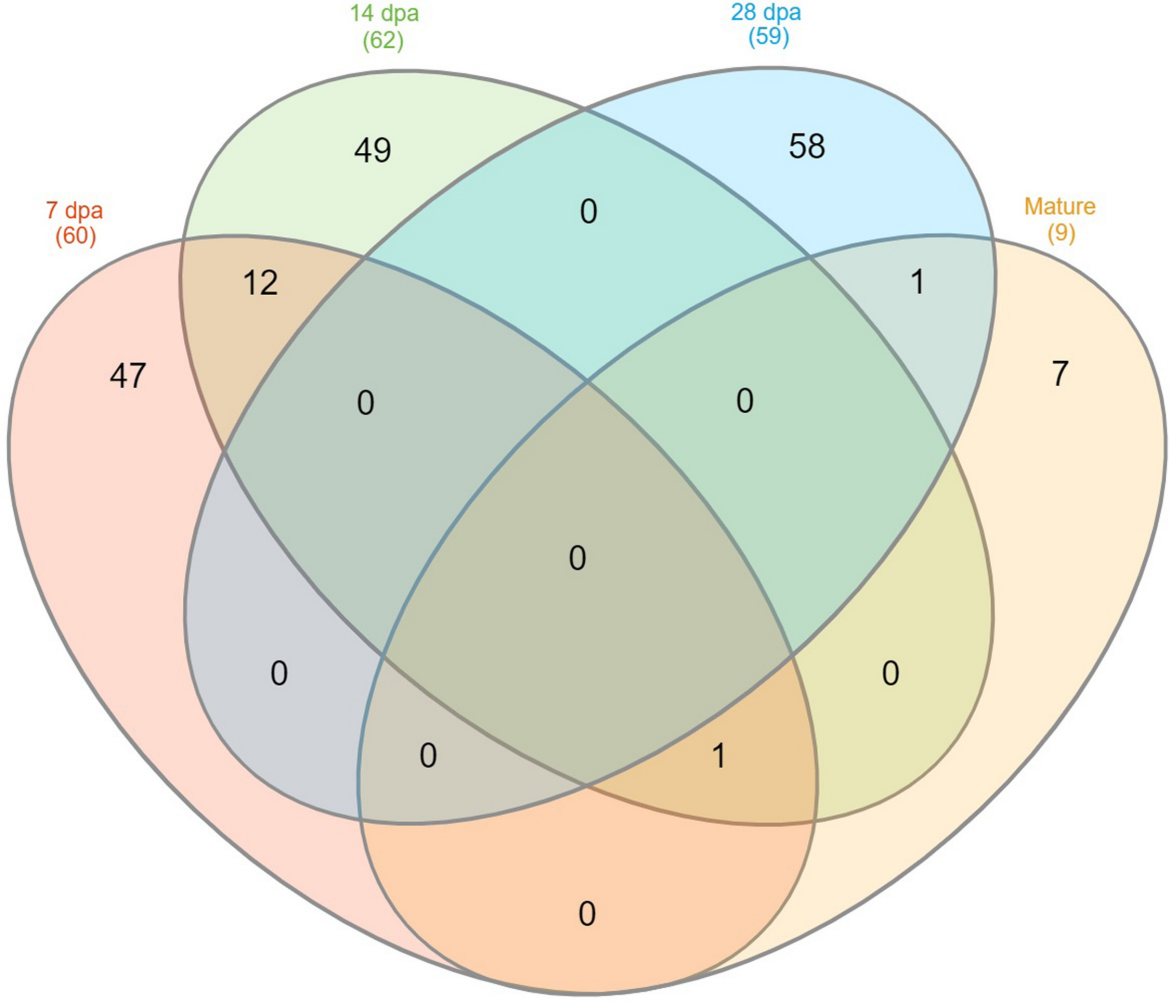
Venn diagram showing the distribution of 175 differentially abundant proteins across different development stages. Differentially abundant proteins are with *P* value ≤ 0.05 and |log2 fold change| ≥ 2. The kernel developmental stages are 7 days post anthesis (dpa), 14 dpa, 28 dpa, and maturity.

Generally, the DAPs vary with the development stages. Most proteomic variation between normal soft and super soft lines was observed at 7 dpa, 14 dpa, and 28 dpa, whereas nine DAPs were identified at maturity (Fig 3 and S3 Table in S1 File).

**Fig 4:**
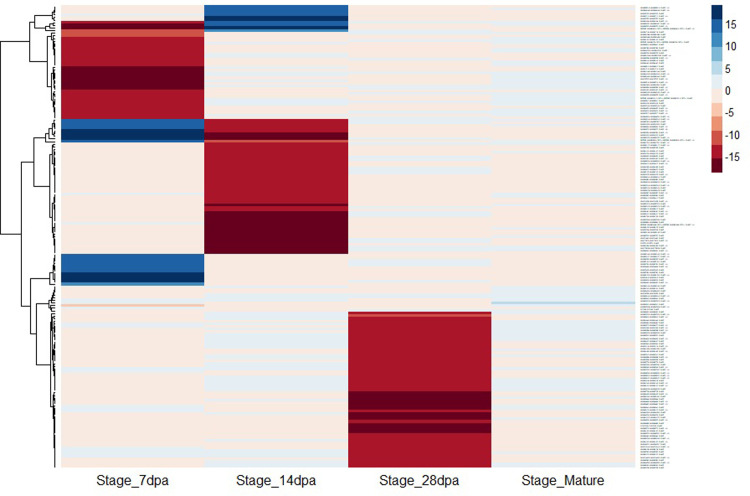
Heat map illustrating log2 fold change of 175 differentially abundant proteins at different development stages. Blue color corresponds to increased abundance in normal soft wheat compared to super soft and red color corresponds to increased abundance in super soft compared to normal soft wheat. The kernel development stages are 7 days post anthesis (dpa), 14 dpa, 28 dpa, and maturity.

Most DAPs were stage-specific and there was no DAP that was observed in all developmental stages. Of the 175 DAPs, 32 had increased abundance in normal soft wheat compared to super soft wheat and were observed at 7 dpa, 14 dpa, and maturity. The remaining 143 DAPs had decreased abundance in normal soft wheat compared to super soft wheat and were mainly observed at 7 dpa, 14 dpa, and 28 dpa (Table 2 and Fig 4). Only 15 proteins were differentially abundant in more than one kernel development stage, however 14 of them had temporal variation as both decreased and increased abundance were observed depending on kernel development stage. For instance, 13 DAPs were identified at 7 dpa and 14 dpa, of which nine had increased abundance at 7 dpa then decreased abundance at 14 dpa and vice versa for the remaining four DAPs (Figs 3 and 4 and S3 Table in S1 File).

**Table 2:** Number of differentially abundant proteins in different kernel development stages. Increased abundance corresponds to higher protein intensity in normal soft wheat compared to super soft wheat whereas, decreased abundance corresponds to lower protein intensity in normal soft wheat compared to super soft wheat.

Stage^a^	Increased abundance	Decreased abundance
**7 dpa**	22	38
**14 dpa**	10	52
**28 dpa**	0	59
**Mature**	7	2
**Total**	32	143

^a^ Kernel developmental stages are 7 days post anthesis (dpa), 14 dpa, 28 dpa, and maturity.

### Gene ontology enrichment analysis

Of the175 DAPs, 135 (77%) were associated with 168 GO terms and the remaining 40 DAPs had no assigned GO terms (S3 Table in S1 File). The 135 GO terms were classified into three categories: biological process (52 GO terms), molecular function (96 GO terms), and cellular component (20 GO terms) (S5 Table in S1 File).

**Fig 5:**
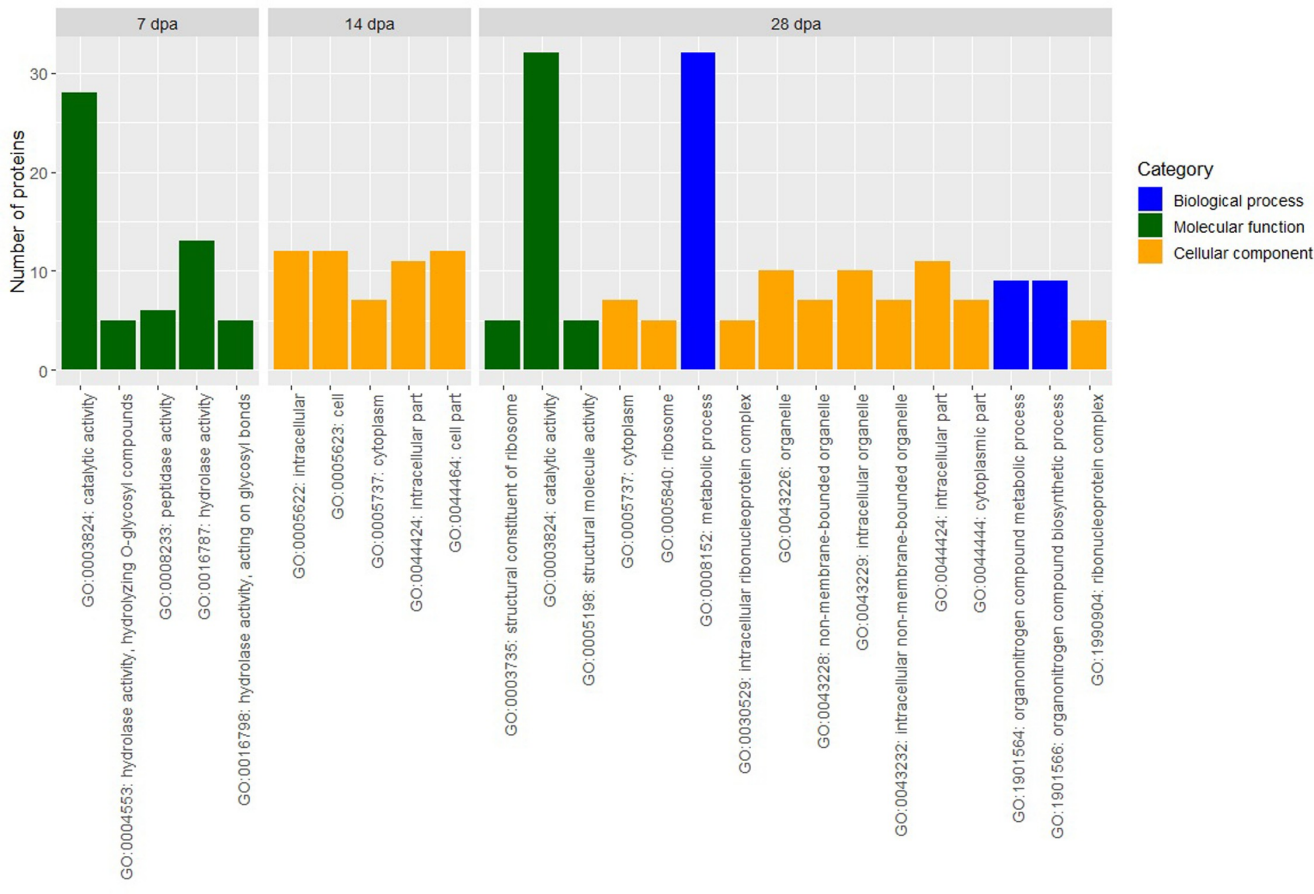
Significant gene ontology (GO) terms from enrichment analysis of differentially abundant proteins at kernel development stages: 7 days post anthesis (dpa), 14 dpa, and 28 dpa.

GO enrichment analysis was performed for DAPs at 7 dpa, 14 dpa, and 28 dpa but not maturity, due to the low number of DAPs at maturity (Fig 5 and S6 Table in S1 File).

We found 23 enriched GO terms associated with 80 DAPs. Enrichment analysis of DAPs at 7 dpa identified five significant GO terms which belong to the category of molecular function. Among these five GO terms, GO:0003824 (catalytic activity) and GO:0016787 (hydrolase activity) had the highest number of DAPs. Enrichment analysis of DAPs at14 dpa, identified five significant GO terms belonging to the category of cellular component. The highest number of enriched GO terms were observed at 28 dpa with 10 GO terms associated with cellular component, three GO terms associated with biological process, and three GO terms associated with molecular function. Among the 16 enriched GO terms at 28 dpa, GO:0003824 (catalytic activity) and GO:0008152 (metabolic process) had the highest number of DAPs. Three significantly enriched GO terms were found to be associated with more than one kernel development stage including GO:0003824 (catalytic activity; 7 dpa and 28 dpa), GO:0044424 (intracellular part; 14 dpa and 28 dpa), and GO:0005737 (cytoplasm; 14 dpa and 28 dpa).

### DAPs associated with significantly enriched gene ontology terms

At 7 dpa, 28 DAPs, associated with five significantly enriched GO terms, were identified (Table 3 and S7 Table in S1 File). Half of these DAPs had increased abundance, whereas the other half had decreased abundance. Among these DAPs, nine were associated with carbohydrate metabolism including UDP-glucose-4-epimerase, glycosyltransferase, alpha-galactosidase, beta-galactosidase, hexosyltransferase, alpha/beta-hydrolases superfamily protein, glycosyltransferase, non-lysosomal glucosylceramidase, and alpha-amylase. Five of these carbohydrate metabolism-associated DAPs had higher abundance in super soft wheat.

**Table 3:** Differentially abundant proteins associated with significantly enriched gene ontology terms at seven days post anthesis.

Accession number	Gene	IWGSC gene annotation ^a^	Chromosome	*T*. *aestivum* RefSeq v1.0 position (Mb) ^b^	Abundance ^c^
**A0A3B5XX46**	*TraesCS1A01G124200*.*1*	Peroxidase	1A	147	Increased
**A0A3B5Y0N7**	*TraesCS1A01G198400*.*1*	ATP-dependent Clp protease proteolytic subunit	1A	356	Increased
**A0A3B5Y3W8**	*TraesCS1A01G327000*.*1*	tRNA pseudouridine synthase d, putative	1A	517	Increased
**A0A3B5Y6U5**	*TraesCS1A01G404600*.*1*	Subtilisin-like protease	1A	568	Increased
**A0A3B5Z677**	*TraesCS1B01G478400*.*1*	UDP-glucose-4-epimerase	1B	687	Decreased
**A0A3B6AVV4**	*TraesCS2A01G185500*.*1*	Tubulin alpha chain	2A	147	Decreased
**A0A3B6B5I5**	*TraesCS2A01G478900*.*1*	Glycosyltransferase	2A	717	Decreased
**A0A3B6B544**	*TraesCS2A01G482700*.*1*	tRNA (Guanine(10)-N2)-methyltransferase-like protein	2A	719	Increased
**A0A3B6CD88**	*TraesCS2B01G491000*.*1*	Polyphenol oxidase	2B	688	Decreased
**A0A1D5V2V1**	*TraesCS2D01G105500*.*3*	Alpha-galactosidase	2D	57	Decreased
**A0A3B6DGC8**	*TraesCS2D01G338700*.*2*	Protein-L-isoaspartate O-methyltransferase	2D	432	Increased
**A0A3B6EIW9**	*TraesCS3A01G241300*.*1*	Pterin-4-alpha-carbinolamine dehydratase	3A	454	Increased
**A0A3B6GXR9**	*TraesCS3D01G260500*.*1*	Chitinase	3D	363	Decreased
**A0A3B6GYZ7**	*TraesCS3D01G379300*.*2*	Beta-galactosidase	3D	496	Increased
**A0A3B6JM18**	*TraesCS4D01G220200*.*1*	Imidazole glycerol phosphate synthase subunit HisF	4D	375	Decreased
**A0A3B6JNG4**	*TraesCS4D01G248100*.*2*	Mitochondrial-processing peptidase beta subunit	4D	417	Decreased
**REVERSE_A0A3B6KC24-DECOY**	*TraesCS5A01G067100*.*1*	Hexosyltransferase	5A	75	Decreased
**A0A3B6KE98**	*TraesCS5A01G074800*.*3*	Translation initiation factor eIF-2B subunit epsilon	5A	88	Decreased
**A0A3B6LTW1**	*TraesCS5B01G412100*.*1*	Alpha/beta-hydrolases superfamily protein	5B	587	Increased
**A0A3B6MXH0**	*TraesCS5D01G373900*.*1*	Molybdenum cofactor sulfurase protein-like	5D	447	Decreased
**A0A3B6MYA2**	*TraesCS5D01G409600*.*1*	Subtilisin-like protease	5D	473	Increased
**A0A3B6NRL0**	*TraesCS6A01G238500*.*1*	Vacuolar-processing enzyme	6A	448	Decreased
**A0A3B6RDS5**	*TraesCS7A01G122900*.*1*	Peroxyureidoacrylate/ureidoacrylate amidohydrolase RutB	7A	80	Increased
**A0A3B6SBI7**	*TraesCS7B01G102500*.*1*	Glycosyltransferase	7B	117	Decreased
**A0A3B6SCZ8**	*TraesCS7B01G143300*.*1*	Succinyl-diaminopimelate desuccinylase	7B	184	Decreased
**A0A3B6TKW0**	*TraesCS7D01G316700*.*1*	Non-lysosomal glucosylceramidase	7D	403	Increased
**A0A3B6TUQ0**	*TraesCS7D01G380400*.*1*	Alpha-amylase	7D	493	Increased
**A0A3B6TS62**	*TraesCS7D01G519000*.*1*	Cytosine-specific methyltransferase	7D	617	Increased

^a^ International Wheat Genome Sequencing Consortium (IWGSC).

^b^ Physical positions of annotated genes based on the Wheat Chinese Spring RefSeq v1.0 genome in mega base pairs (Mb).

^c^ Increased abundance corresponds to higher abundance in normal soft wheat compared to super soft wheat, whereas decreased abundance corresponds to lower abundance in normal soft wheat compared to super soft wheat.

At 14 dpa, there were 11 DAPs belonging to five significant GO terms that were mainly associated with regulation and signaling (Table 4 and S7 Table in S1 File). Nine of these DAPs had a higher abundance in super soft wheat.

**Table 4:** Differentially abundant proteins associated with significantly enriched gene ontology terms at 14 days post anthesis.

Accession number	Gene	IWGSC gene annotation ^a^	Chromosome	*T*. *aestivum* RefSeq v1.0 position (Mb) ^b^	Abundance ^c^
**A0A3B5Y185**	*TraesCS1A01G214600*.*1*	10 kDa chaperonin	1A	377	Decreased
**A0A3B5ZVF5**	*TraesCS1D01G228700*.*1*	Ubiquitin	1D	317	Increased
**A0A3B6AW16**	*TraesCS2A01G203700*.*1*	Elongator complex protein 1	2A	180	Decreased
**A0A3B6AWJ4**	*TraesCS2A01G218600*.*1*	PPDK regulatory protein	2A	205	Decreased
**A0A3B6BY61**	*TraesCS2B01G053100*.*4*	Eukaryotic translation initiation factor 3 subunit M	2B	26	Decreased
**A0A3B6GUN5**	*TraesCS3D01G248700*.*4*	Myosin	3D	348	Decreased
**A0A3B6GZF5**	*TraesCS3D01G392700*.*2*	Nuclear transport factor 2 family protein with RNA binding domain	3D	508	Decreased
**A0A3B6KRE7**	*TraesCS5A01G442200*.*2*	Lysine—tRNA ligase	5A	623	Decreased
**A0A3B6LRN5**	*TraesCS5B01G384400*.*1*	DNA repair protein XRCC4	5B	563	Decreased
**A0A3B6MYA0**	*TraesCS5D01G449600*.*1*	Lysine—tRNA ligase	5D	498	Decreased
**A0A3B6PQC8**	*TraesCS6B01G290800*.*1*	AP-2 complex subunit mu	6B	523	Decreased

^a^ International Wheat Genome Sequencing Consortium (IWGSC).

^b^ Physical positions of annotated genes based on the Wheat Chinese Spring RefSeq v1.0 genome in mega base pairs (Mb).

^c^ Increased abundance corresponds to higher abundance in normal soft wheat compared to super soft wheat, whereas decreased abundance corresponds to lower abundance in normal soft wheat compared to super soft wheat.

At 28 dpa, there were 41 DAPs, associated with 16 significant GO terms and having higher abundance in super soft wheat (Table 5 and S7 Table in S1 File). Six of these proteins were associated with carbohydrate metabolism including, cell wall invertase, pectin lyase-like superfamily protein, endoglucanase, sucrose-phosphate synthase, acetyltransferase component of pyruvate dehydrogenase complex, and starch synthase, whereas two them were lipid-related including, TraesCS4A01G397000.1 (GDSL esterase/lipase) and TraesCS7A01G113600.1 (GDSL esterase/lipase).

**Table 5:** Differentially abundant proteins associated with significantly enriched gene ontology terms at 28 days post anthesis.

Accession number	Gene	IWGSC gene annotation ^a^	Chromosome	*T*. *aestivum* RefSeq v1.0 position (Mb) ^b^	Abundance ^c^
**A0A3B5Y266**	*TraesCS1A01G241900*.*1*	UDP-sulfoquinovose synthase	1A	430	Decreased
**A0A3B5Y7U4**	*TraesCS1A01G433800*.*1*	50S ribosomal protein L16	1A	585	Decreased
**A0A3B6AT43**	*TraesCS2A01G092400*.*1*	D-alanine—D-alanine ligase	2A	45	Decreased
**A0A3B6AS45**	*TraesCS2A01G099500*.*1*	Subtilisin-like protease	2A	53	Decreased
**A0A3B6ARN8**	*TraesCS2A01G102500*.*1*	NADH-cytochrome b5 reductase	2A	56	Decreased
**A0A3B6AX60**	*TraesCS2A01G210900*.*1*	50S ribosomal protein L23	2A	195	Decreased
**A0A3B6C6X0**	*TraesCS2B01G311900*.*1*	Cell wall invertase	2B	446	Decreased
**A0A3B6CAL5**	*TraesCS2B01G416900*.*1*	Subtilisin-like protease	2B	596	Decreased
**A0A3B6CG34**	*TraesCS2B01G576100*.*2*	Calreticulin-like protein	2B	765	Decreased
**A0A3B6CG81**	*TraesCS2B01G579900*.*1*	Lecithin-cholesterol acyltransferase-like 1	2B	768	Decreased
**A0A1D5UXM8**	*TraesCS2D01G060100*.*3*	Cleavage and polyadenylation specificity factor subunit 1	2D	25	Decreased
**A0A3B6DFB4**	*TraesCS2D01G327700*.*1*	Peroxidase	2D	421	Decreased
**A0A3B6DI05**	*TraesCS2D01G402100*.*1*	Pectin lyase-like superfamily protein	2D	517	Decreased
**A0A3B6EIS8**	*TraesCS3A01G191700*.*4*	Magnesium-protoporphyrin IX monomethyl ester [oxidative] cyclase	3A	244	Decreased
**A0A3B6HNX1**	*TraesCS4A01G050800*.*1*	2-oxoglutarate-dependent dioxygenase-related family protein	4A	42	Decreased
**A0A3B6HT09**	*TraesCS4A01G061900*.*1*	Allene oxide synthase	4A	59	Decreased
**A0A3B6HT75**	*TraesCS4A01G091100*.*1*	50S ribosomal protein L4	4A	98	Decreased
**A0A3B6HXN3**	*TraesCS4A01G248200*.*1*	Endoglucanase	4A	559	Decreased
**A0A3B6I0H0**	*TraesCS4A01G397000*.*1*	GDSL esterase/lipase	4A	672	Decreased
**A0A3B6I1P3**	*TraesCS4A01G420600*.*1*	Methyltransferase, putative	4A	691	Decreased
**A0A3B6IMZ2**	*TraesCS4B01G091100*.*1*	Sucrose-phosphate synthase	4B	94	Decreased
**A0A3B6JJ89**	*TraesCS4D01G151500*.*3*	30S ribosomal protein S5	4D	177	Decreased
**A0A3B6JPU8**	*TraesCS4D01G289300*.*2*	Adenylosuccinate synthetase	4D	460	Decreased
**A0A3B6KG09**	*TraesCS5A01G184900*.*1*	protein kinase family protein	5A	385	Decreased
**A0A3B6KLP1**	*TraesCS5A01G300400*.*1*	Cysteine synthase	5A	510	Decreased
**A0A3B6KMP6**	*TraesCS5A01G411500*.*1*	Long-Chain Acyl-CoA Synthetase	5A	600	Decreased
**A0A3B6LG94**	*TraesCS5B01G038800*.*1*	Nucellin-like aspartic protease	5B	44	Decreased
**A0A3B6NQE7**	*TraesCS6A01G210500*.*1*	Cytochrome P450	6A	382	Decreased
**A0A3B6NTC2**	*TraesCS6A01G286600*.*1*	Ribosomal RNA small subunit methyltransferase b	6A	519	Decreased
**A0A3B6KHD5**	*TraesCS6A01G389300*.*1*	Protein kinase family protein	6A	605	Decreased
**A0A3B6PK40**	*TraesCS6B01G200800*.*1*	30S ribosomal protein S21	6B	240	Decreased
**A0A3B6QBJ3**	*TraesCS6D01G024400*.*1*	Acetyltransferase component of pyruvate dehydrogenase complex	6D	9	Decreased
**A0A3B6RDB9**	*TraesCS7A01G111300*.*1*	Mitogen-activated protein kinase	7A	68	Decreased
**A0A3B6RDH5**	*TraesCS7A01G113600*.*1*	GDSL esterase/lipase	7A	70	Decreased
**A0A3B6RFG9**	*TraesCS7A01G172600*.*1*	Alpha/beta-hydrolases superfamily protein, putative	7A	127	Decreased
**A0A3B6RG03**	*TraesCS7A01G301900*.*1*	DNA-directed RNA polymerase subunit	7A	422	Decreased
**A0A3B6RSD0**	*TraesCS7A01G534500*.*1*	WD40 repeat-like protein	7A	712	Decreased
**A0A3B6SIC5**	*TraesCS7B01G270500*.*1*	Acid phosphatase 1	7B	496	Decreased
**A0A3B6TB55**	*TraesCS7D01G160700*.*1*	Nucleolar GTP-binding protein 1	7D	111	Decreased
**Q2WGB1**	*TraesCS7D01G190100*.*1*	Starch synthase, chloroplastic/amyloplastic	7D	144	Decreased
**A0A3B6TTK9**	*TraesCS7D01G328300*.*2*	Alcohol dehydrogenase, putative	7D	420	Decreased

^a^ International Wheat Genome Sequencing Consortium (IWGSC).

^b^ Physical positions of annotated genes based on the Wheat Chinese Spring RefSeq v1.0 genome in mega base pairs (Mb).

^c^ Increased abundance corresponds to higher abundance in normal soft wheat compared to super soft wheat, whereas decreased abundance corresponds to lower abundance in normal soft wheat compared to super soft wheat.

## Discussion

Grain hardness is a key factor of wheat end-use quality. Super soft kernel texture represents an opportunity to improve soft wheat milling and baking quality [11–13, 15]. To enhance our understanding of the mechanism underlying super soft kernel phenotype, we investigated the proteomic variation between normal soft and super soft wheat throughout kernel development. As the parents of the studied lines are genetically related (87.5% identical), Many of DAPs in this study should be associated with variation in kernel texture. Among the 175 DAPs in our study, none were associated with the puroindolines because wild-type puroindoline genes were fixed in all tested genotypes. In addition to the puroindolines, the storage proteins, i.e., albumins, globulins, gliadins, and glutenins are major protein components of wheat kernel [36]. However, the storage proteins were not among the DAPs in this study.

Most of the protein variation between normal soft and super soft wheat was observed within the first four weeks post anthesis (7, 14, and 28 dpa). We also found that most of the DAPs were stage specific. Wheat grain development involves three distinct phases which are cell division and differentiation, grain filling, and desiccation/maturation [37]. Therefore, protein expression is expected to vary through grain developmental stages. Wheat proteins that were developmental stage-specific were reported in previous studies [38, 39].

It was reported that grain hardness is affected by the interaction between carbohydrates and proteins [2, 40]. There are two major types of proteins which are associated with starch granules, storage proteins and starch granule-associated proteins [2]. The storage proteins glutenins and gliadins, which are adsorbed to starch granules, were not among DAPs in this study. The 18 DAPs between normal soft and super soft wheat that are associated with carbohydrate metabolism could have played a role in changing starch-protein interactions, however further biochemical evidence is needed. Grain endosperm is composed of starch (80–85% of dry grain mass) that is synthesized and accumulated during grain filling [41]. While starch synthase enzymes synthesize starch by mobilizing products of sucrose metabolism to the grain, amylases degrade starch to provide energy. The products of sucrose breakdown are essential for starch biosynthesis [42]. Sucrose can be either cleaved by sucrose synthase to produce UDP-glucose and fructose or by invertase to produce glucose and fructose [43]. In our study TraesCS2A01G036100.1 (invertase), TraesCS2B01G311900.1 (cell wall invertase), TraesCS4B01G091100.1 (sucrose-phosphate synthase), and TraesCS7D01G190100.1 (starch synthase) had higher abundance in super soft wheat at 28 dpa. On the other hand, TraesCS7D01G380400.1 (alpha-amylase) had higher abundance in normal soft wheat at 7 dpa. This suggests that starch accumulation could be higher in super soft wheat compared to normal soft wheat. These results also suggest that the mechanics of starch biosynthesis occur along a different timeline in the super soft vs. normal soft lines. Therefore, it is possible that the amount and type of starch synthesized could impact grain hardness. Further, sucrose-phosphate synthase was decreased in abundance at 28 dpa, plausibly this could indicate either a decrease in the unloading of sucrose during grain fill or slowing in the rate of sucrose metabolism to provide glucose for starch biosynthesis.

The degree of adhesion between starch granules and protein matrix was reported to be associated with wheat grain hardness. For instance, soft wheat has lower adhesion between starch and protein compared to hard wheat [2, 44, 45]. In addition, Barlow et al. [44] reported that the interaction between starch granules and amyloplast membranes differs between hard wheat and soft wheat. Glenn and Saunders [46] also found that intracellular space around the starch granules exists in soft wheat, but not hard wheat, which results in a discontinuity in the starch-protein matrix. These previous studies focused on the differences in starch properties between soft and hard wheat, however the relationship between starch characteristics and super soft kernel texture is not yet elucidated. Further research to analyze starch in normal soft and super soft wheat will help understand the effect of the identified carbohydrate metabolism-associated DAPs on grain hardness.

Lipids can be inside starch granules, on the surface of starch granules, or not associated with starch [10]. Grain hardness is also associated with the content of polar lipids (glycolipids and phospholipids) present on the starch surface as they may weaken the starch: protein matrix interaction [47, 48]. In this study, lipid metabolism proteins were found to be higher in super soft wheat compared to normal soft wheat, especially at 28 dpa. Kim et al. [49] reported that soft kernel genotypes contain more polar lipids than hard kernel genotypes, with the differences increasing throughout kernel development. Similarly, Feiz et al. [50] found that softer kernel genotypes had increased polar lipids. In another study, Morrison et al. [51] found that the decreasing amount of free polar lipids is strongly correlated with increasing grain hardness in some wheat cultivars. Further research to analyze lipids can explain the role of the five identified lipid-associated proteins in decreasing grain hardness.

The comparisons between the genomic positions of large effect quantitative trait loci (QTL) associated with super soft kernel texture in previous studies and the corresponding encoding genes of DAPs in this study showed three co-localized loci. For instance, the gene *TraesCS1B02G478400*.*1* (UDP-glucose-4-epimerase, 687 Mb, 7 dpa, decreased abundance) was located close to single nucleotide polymorphism (SNP) markers, *GBS_492*, *GBS_498*, and *GBS_499* which were located at 650–652 Mb (based on the durum wheat cv. Svevo genome v1; [52] and were associated with super soft phenotype in durum wheat [16]. These previously identified SNP markers in durum wheat explained between 10% and 12% of the phenotypic variation in grain hardness. Similarly, *TraesCS5A01G184900*.*1* (protein kinase family protein, 385 Mb, 28 dpa, decreased abundance) was positioned close to SNP markers associated with super soft phenotype in white winter wheat including *S5A_389917237* (390 Mb), *S5A_389917278* (390 Mb), and *S5A_396230360* (396 Mb) [15]. Further, *TraesCS4B02G091100*.*1* (sucrose-phosphate synthase, 94 Mb, 28 dpa, decreased abundance) was located close to the position of the major QTL on chromosome arm 4BS linked to the SNP marker, *Excalibur_c100336_106* (86 Mb) and associated with super soft phenotype in BC2SS163 [12]. Kumar et al. [12] identified a large-effect QTL on chromosome 4BS (designated as *Qssoft*.*wwql-4B*.*1*) in BC2SS163, explaining 36.7% of phenotypic variation and with a logarithm of odds (LOD) score of 21.7. Given the genomic position of *TraesCS4B02G091100*.*1* and the function of sucrose synthase in sucrose catabolism, *TraesCS4B02G091100*.*1* on chromosome 4BS is likely a candidate gene for the super soft kernel texture in the white spring wheat line BC2SS163. To validate the effect of *TraesCS4B02G091100*.*1* on grain hardness, measuring sucrose synthase activities, protein content (using immunoblotting), and transcript levels is warranted.

## Conclusions

Proteomic analysis identified 175 differentially abundant proteins (DAPs) that were mainly identified at 7, 14, and 28 dpa. Because the parents of the RILs are closely related, most DAPs should be associated with variation in grain texture. Grain hardness is known to be affected by the interaction between carbohydrates, proteins, and lipids, thus the identified 23 DAPs associated with carbohydrate metabolism and lipids in this study could be related to kernel texture. The manipulation of these proteins could lead to softer kernels and thus improved end-use quality in soft wheat. GO enrichment analysis identified 23 significantly enriched GO terms, of which GO:0003824 (catalytic activity) and GO:0008152 (metabolic process) had the highest number of DAPs. Based on previous genetic analysis study of super soft kernel texture in BC2SS163 and the current study, *TraesCS4B02G091100*.*1* which encodes for sucrose-phosphate synthase is likely a candidate gene associated with the super soft kernel phenotype in BC2SS163. Further validation of the effect of *TraesCS4B02G091100* on grain hardness is worth exploring in future studies.

## Supporting information

S1 File (XLSX)
